# An Acoustic Sensor for Combined Sewer Overflow (CSO) Screen Condition Monitoring in a Drainage Infrastructure

**DOI:** 10.3390/s21020404

**Published:** 2021-01-08

**Authors:** Chan H. See, Kirill V. Horoshenkov, M. Tareq Bin Ali, Simon J. Tait

**Affiliations:** 1 School of Engineering and the Built Environment, Edinburgh Napier University, Edinburgh EH10 5DT, UK; 2 Pennine Water Group, University of Sheffield, Sheffield S1 3JD, UK; k.horoshenkov@sheffield.ac.uk (K.V.H.); m.tareq@gmail.com (M.T.B.A.); s.tait@sheffield.ac.uk (S.J.T.)

**Keywords:** CSO, acoustic sensor, wastewater infrastructure, screen

## Abstract

Combined sewer overflow structures (CSO) play an important role in sewer networks. When the local capacity of a sewer system is exceeded during intense rainfall events, they act as a “safety valve” and discharge excess rainfall run-off and wastewater directly to a natural receiving water body, thus preventing widespread urban flooding. There is a regulatory requirement that solids in CSO spills must be small and their amount strictly controlled. Therefore, a vast majority of CSOs in the UK contain screens. This paper presents the results of a feasibility study of using low-cost, low-energy acoustic sensors to remotely assess the condition of CSO screens to move to cost-effective reactive maintenance visits. In situ trials were carried out in several CSOs to evaluate the performance of the acoustic sensor under realistic screen and flow conditions. The results demonstrate that the system is robust within ±2.5% to work successfully in a live CSO environment. The observed changes in the screen condition resulted in 8–39% changes in the values of the coefficient in the proposed acoustic model. These changes are detectable and consistent with observed screen and hydraulic data. This study suggested that acoustic-based sensing can effectively monitor the CSO screen blockage conditions and hence reduce the risk of non-compliant CSO spills.

## 1. Introduction

In the UK, there is over 600,000 km of sewer pipes and over 70% of these are combined, in that they carry both wastewater and rainfall run-off from urban surfaces [1]. Due to climate change and increasing capacity demand because of increased population and urbanization, this ageing infrastructure is progressively under pressure and deteriorating, causing a steady increase in maintenance budgets [2,3]. 

Water companies are regulated by the Office of Water Services (OFWAT), which is a non-ministerial government department that is responsible for ensuring that water companies offer adequate service to the customers at a fair price [2]. With these stringent regulations, water companies have invested more than £8 billion in 2019–20 to improve their level of services. These include customers’ sewer repair, replacement and rehabilitation, one of the major areas of spend [3]. This has opened an unprecedented opportunity for instrument makers and researchers to develop partnerships to offer novel approaches to proactively reduce the risk of failure and allow better understanding of the behaviour of the sewer network under both normal and extreme operating conditions [4,5,6,7,8,9,10,11,12].

There are approximately around 31,000 combined sewer overflows (CSOs) in the UK [13]. CSOs play an important role as emergency discharge structures in a sewer network. Their primary function is to limit the discharge of wastewater to wastewater treatment plants, without causing local flooding when the sewerage system is overloaded by intense rainfall. When the capacity of the sewer system comes close to its maximum, excess flow is discharged directly into a local watercourse. These discharges are regulated by the Environment Agency, and water companies are required to comply with aesthetic control standards, which ensure that no solids having dimension greater than 6 mm in two orthogonal directions can be released [14]. Water companies have therefore installed screens within the CSO chamber to meet the aesthetic control standards. The condition of the CSO screen determines the ability of a CSO to operate to its legal discharge consent. Blocked screens can lead to CSO discharges being bypassed and CSO spills containing solids that do not meet the aesthetic control standards and leave them liable to substantial financial penalties. In order to reduce the risk of screens failing, they need to be regularly inspected and maintained (cleaned). This is costly for water utilities as planned maintenance requires engineers to visit a large number of remote sites, to potentially institute traffic management, enter a confined space (CSO chamber) and then manually inspect the screen conditions. Screens get blocked after spill events, so a preplanned maintenance program often results in many unnecessary visits. A more cost-efficient and safe approach would be to acquire the real-time information about the screen condition if it is required to be cleaned.

**Figure 1:**
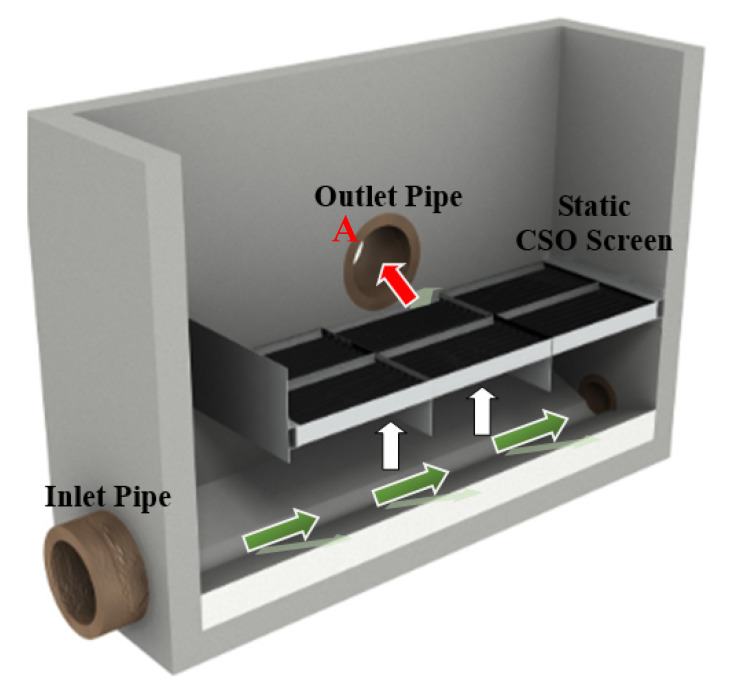
Sectional internal view of a traditional combined sewer overflow (CSO) chamber (adapted from [15]).

The key physical assets that constitute a CSO are the inlet and outlet pipes, which convey wastewater downstream. The diameter of these pipes, their slope and wastewater discharge determine the water level in the CSO chamber. If the wastewater level exceeds a certain threshold, e.g., in the case of a heavy rainstorm event or downstream blockage, then wastewater water spills through the CSO screen into the outlet pipe, into a receiving watercourse, e.g., local river or natural stream. The screen is installed so that spill flow can be screened for large solids. If the screen gets blocked, then an emergency overflow can bypass the screen (marked A in Figure 1) to allow unscreened wastewater to be directly discharged to the receiving watercourse. In the latter case, the CSO is failing its discharge consent.

To monitor the conditions of the inlet and outlet pipes of the CSO, several pipe inspection methods have had either destructive or non-destructive testing approaches proposed and implemented in the literature [16,17,18,19,20,21,22,23]. Currently, closed circuit television (CCTV) methods [16,17] are one of the most common ways to survey the pipe. Several fast and efficient alternative methods to analyse the condition of a sewer pipe wall objectively were proposed [8,10,18,19,20,21,22,23]. These methods are acoustic [8,10,18], acoustic optical [19], electromagnetic [20,21], electroacoustic [22], ultrasonic [5,7,23] and laser-based [8] methods. These methods are not designed to work in CSOs. The only sensors which are installed in CSO are ultrasonic water level meters [24,25] and optical fibre-based condition monitoring systems [26]. The ultrasonic water level sensor monitors the water levels to inform the operator the frequency of spill incidents when water level is high enough to be discharged via the outlet pipe as shown in Figure 1, while the fibre optical sensor is used to detect the changes of humidity and temperatures. However, none of these methods is suitable for the analysis of CSO screen conditions. Therefore, there is little or no knowledge about the propensity of the CSO to discharge unscreened wastewater into the receiving watercourse.

Therefore, there is a clear need for a new instrument to continuously monitor the real-time condition of CSO screens. This technology will present the opportunity for screens to be cleaned before or shortly after they have become seriously blinded, preventing sewage debris from hardening and ensuring that they are clean and able to pass sufficient flow before the next wastewater discharge event. This also will ensure that the screen is operating at its design performance at all times, hence reducing the risk of unconsented spills. This paper describes the operation principle and six-month-long field experiments with a new low-cost acoustic sensor that is able to access rapidly the condition of a static screen. The data provided by this type of sensor will enable water companies to manage their sewerage infrastructure at a significantly lower cost by proactively responding to the potential of CSO events. 

The structure of the remainder of this paper can be summarised as follows. In Section 2, the details of the system architecture and the design methodology are presented. The results from the two phases of field trials, i.e., short-term and longer term, for the proposed acoustic instrument are discussed in Section 3. Finally, conclusions are drawn in Section 4.

## 2. Materials and Methods

### 2.1. System Architecture and Design Methodology

Figure 1 shows the structure and working principle of a CSO. Under dry weather flow conditions, wastewater flows in the direction of the green arrow direction underneath the screen and downstream of the CSO. In a storm event, the water level will rise within the chamber. If the storm is intense or long enough, then the water level can reach the weir crest to spill through the screen (see the white arrows). The unwanted solids and floatables are trapped on the lower surface of the screen while wastewater carrying a reduced amount of solids is discharged through the outlet pipe into the receiving watercourse (see red arrow).

**Figure 2:**
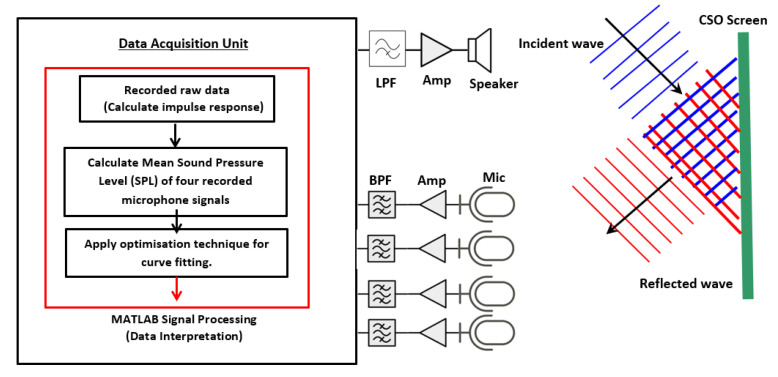
Basic operation block diagram of the system.

In order to monitor the condition of a CSO screen, an acoustic measurement system is proposed. The general system architecture of this system is described in Figure 2. The system consists of four microphones, speaker, temperature sensor and data acquisition unit. The speaker is connected to a power amplifier and a low-pass filter (LPF) with a cut-off frequency of 1000 Hz. Each of the four microphones is integrated with an amplifier and a band pass filter (BPF). This will ensure that the right spectrum of the excited signal is emitted and the desired frequency band of output signal is recorded in the frequency range between 100 and 1000 Hz. The system is battery operated, but it can be adapted to work with an energy harvesting system (e.g., solar panel or wind turbine). For the safety of operation, as methane gas may be produced or other volatile vapours (from fuel spills) may be present in the CSO chamber, Zener diode barriers were installed to prevent the system from any surge in electrical current incident leading to a spark.

This proposed system is based on an acoustic approach [18,19] developed for the purpose of sewer inspection and adapted in our work for screen condition monitoring. The method is based on measuring the reverberant sound field in the CSO chamber, which is affected by debris accumulating on the CSO screen. Figure 2 illustrates the block diagram of the proposed approach to better explain the signal processing algorithm adopted in this work. As can be seen, an acoustic sensor is installed in a manhole chamber and a 10 s sinusoidal sweep signal (50 Hz to 7500 Hz) is sent out through the speaker. The reflected signal is then captured by four microphones and sent to the data acquisition unit (DAU) to be digitized and recorded. The acquired data are then stored on a data logger, which is regularly collected for off-line data analysis. 

In the signal analysis process, the recorded four channel microphone acoustic signals are deconvolved to obtain the acoustic impulse response of the chamber, $s_{j}\left(t\right),\;j=1,2,3$, 4 which is filtered through a band pass filter (100 to 1000 Hz) and then converted to the time-dependent sound pressure level (SPL) [18,19]:

(1) $$L_{j}(t_{n})=10\log_{10}\left\{\frac{1}{\tau }\int_{t_{n}}^{t_{n}+\tau }s_{j}^{2}(t)dt\right\}$$

where the integration constant τ=3 msec and $t_{n}=\tau n,\;n=0,\ldots ,N$. The choice of this frequency range can be explained by the fact that the selected speaker was only able to produce sounds efficiently at frequencies above 100 Hz. This dictated the low-frequency limit we adopted. At frequencies above 1000 Hz, the attenuation in the chamber was too high because of the relatively high moisture level in the air. As a result, it was difficult to measure the sound pressure level over a large enough dynamic range at frequencies above 1000 Hz. Therefore, 1000 Hz was adopted as the upper frequency limit in the analysis described in the following text. The direct average of the four SPLs is then computed as [18,19]:

(2) $$\bar{L}\left(t_{n}\right)=\frac{1}{4}\overset{4}{\underset{j=1}{\sum }}L_{j}\left(t_{n}\right)$$

**Figure 3:**
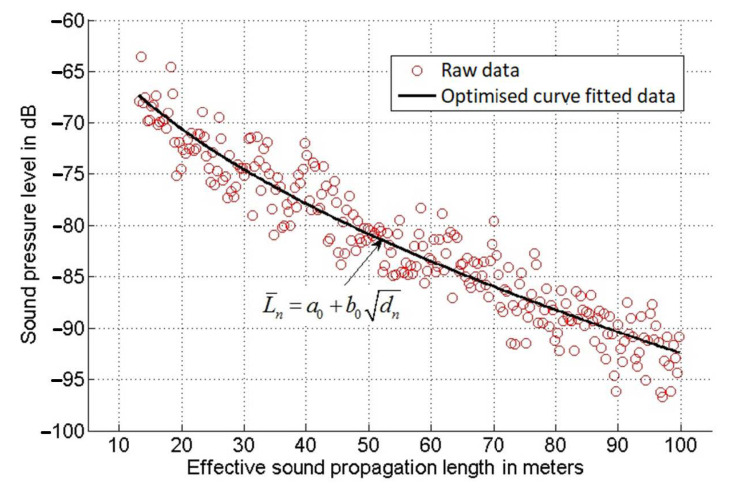
Example of the curve fitting process.

Figure 3 shows a typical mean SPL time history (red circle) as a function of the effective propagation length, $d=C_{air}t$, 15≤d≤100 m, where *C_air_* = (331.3 + 0.606 θ) m/s is the sound speed in air and θ is the temperature in degrees Celsius (°C) [27]. The choice for this range can be explained by the fact that for *d* < 15 m (i.e., below 44 msec at θ = 20 °C), the recorded signal was dominated by the sound which was coming directly from the speaker. This sound was yet to be reflected by the screen multiple times to form a fully diffused sound field to carry useful information on the screen condition. For *d* > 100 m (0.29 s at θ = 20 °C), the acoustic signal was attenuated well below the background noise floor so that it could not be used in the analysis. It was found that a change in the air temperature had little or no effect on the measured sound pressure level time history $\bar{L}\left(d\right)$ for a given screen condition.

In order to simplify the process of analysing the meaning of this graph, a least mean squares curve fitting method [28] was adopted to provide an analytical fit to the recorded sound pressure level data. In this study, the following polynomial equation was used:

(3) $${\bar{L}}_{n}=a_{0}+b_{0}\sqrt{d_{n}}$$

where

(4) $$b_{0}=\frac{\sum \sqrt{d_{n}}{\bar{L}}_{n}-\frac{\sum {\bar{L}}_{n}\sum \sqrt{d_{n}}}{N}}{\left(\sum d_{n}-\frac{\sum \sqrt{d_{n}}\sum \sqrt{d_{n}}}{N}\right)}$$

(5) $$a_{0}=\frac{\sum {\bar{L}}_{n}-b_{0}\sum \sqrt{d_{n}}}{N}$$

Here ${\bar{L}}_{n}$ is the sound pressure level recorded at the time instance *t_n_*, *d_n_* is the distance, *n* is the integer in the time step, *a*_0_ is the SPL level at the intercept point when *d_n_* = 0 and *b*_0_ is the gradient of the curve. This method enabled us to identify the optimal values for the *a*_0_ and *b*_0_ coefficients for the best fit of a set of data. Figure 3 presents an example of experimental data and corresponding polynomial fit obtained using the LMS method.

The coefficients *a*_0_ and *b*_0_ have a clear physical meaning. The slope of the curve (*b*_0_) gives good indication of the conditions of the screen. A smaller absolute value of *b*_0_ indicates a smaller acoustic absorption of the screen, which results in a longer reverberation time in the CSO chamber. This means that the CSO screen condition is cleaner. On the contrary, a larger absolute value of *b*_0_ suggests that the CSO screen becomes contaminated with organic wastewater porous debris which absorbs sound better. This is an indicator that the screen is getting blinded. In this case, the acoustic attenuation in the chamber increases and so does the reverberation time. The coefficient *a*_0_ corresponds to the intercept point of the SPL curve when *d* is zero. This coefficient changes consistently with a change in *b*_0_ and it is an estimate of the maximum sound pressure level in the CSO. It is controlled by the power of the sound radiated by the speaker, the absorption area of the screen and walls in the CSO chamber and its volume. 

### 2.2. Field Trials

There were two phases of field trial for the proposed acoustic instrument. Phase 1 involved two one-day experiments in two real CSO chambers with two static screens with dimensions 820 × 3380 mm^2^ and 2300 × 4125 mm^2^. The findings obtained from these tests were used to inform the second phase tests. Phase 2 was to investigate the feasibility of in situ implementation of the proposed sensor within the CSO chamber with a screen size of 505 × 1480 mm^2^ over a period of six months. It should be noted that a typical CSO screen is a simple porous structure which is basically a stainless steel mesh. It consists of a 6 × 6 mm profile wedge wire to provide a larger surface area to enable a spill of a sufficient flow of wastewater filtering out any larger debris.

## 3. Results

### 3.1. Phase 1 Short-Term Experiments and Results

**Figure 4:**
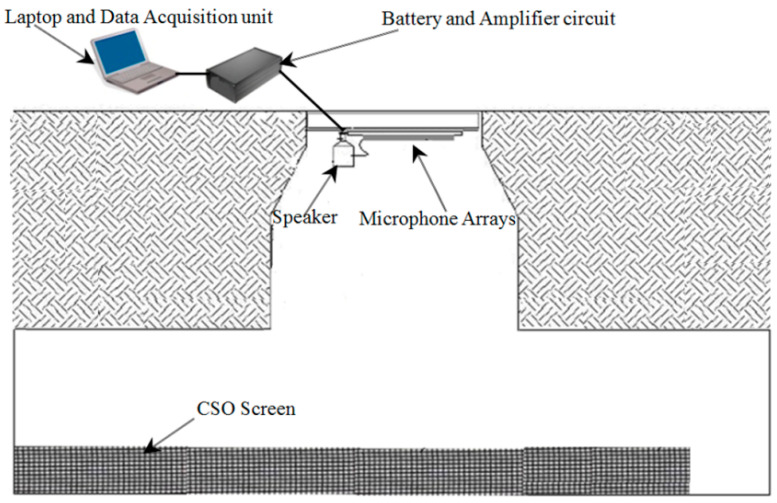
Installation diagram of the acoustic sensor in the chamber.

**Figure 5:**
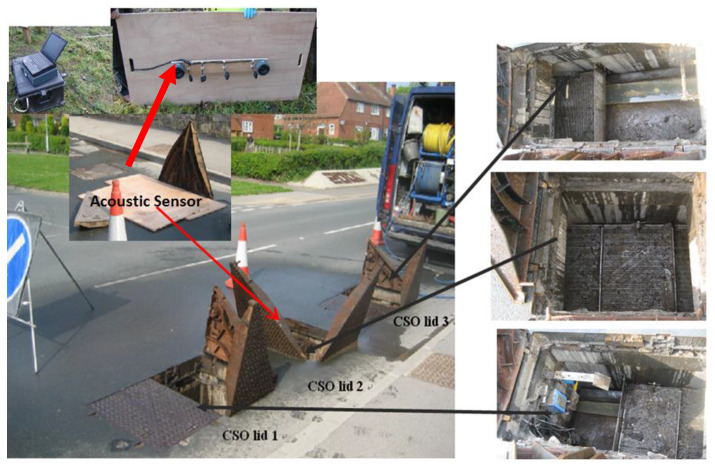
Field trial on site 1 at a CSO chamber in an urban area in Yorkshire.

For phase 1 tests, an intrinsically safe version of the existing laboratory acoustic instrument was developed. This system consisted of four microphone arrays and a speaker, which were fastened on a wood panel, an IBM Lenovo Thinkpad tablet, a PCMCIA sound card (model: Digigram VXPocket 440) and a box of batteries with an amplifier circuit. This system and the method for its installation in phase 1 are shown in Figure 4. The system was initially tested in the laboratory prior to field trial. The acoustic instrument was then installed temporarily in an existing live CSO chamber. Figure 5 presents photographs of one of the two CSO sites and arrangements for the static screens. Four screen conditions were tested in these experiments: original unclean, first level of cleaning, second level of cleaning and fully clean. The unclean condition corresponded to a heavily blocked screen, which was found when the CSO chamber was opened. The first and second levels of cleaning conditions were simulated by using high pressure water jetting to gradually reduce the blocked condition in controlled portions of the CSO screen. Effectively, this means that in the field trial, we progressively cleaned the amount of debris blocking the screen perforations. This enabled us to simulate in the field four screen conditions from fully blocked condition to partially blocked condition and, finally, to fully cleaned condition. In every condition, the screen was photographed and an acoustic measurement was taken until all the debris was removed to a desired level. This would enable the acoustic attenuation data for a known percentage of screen contamination to be obtained to validate the proposed technology and calibrate the acoustic response of the CSO chamber against visual images of the condition of the CSO screen.

**Figure 6:**
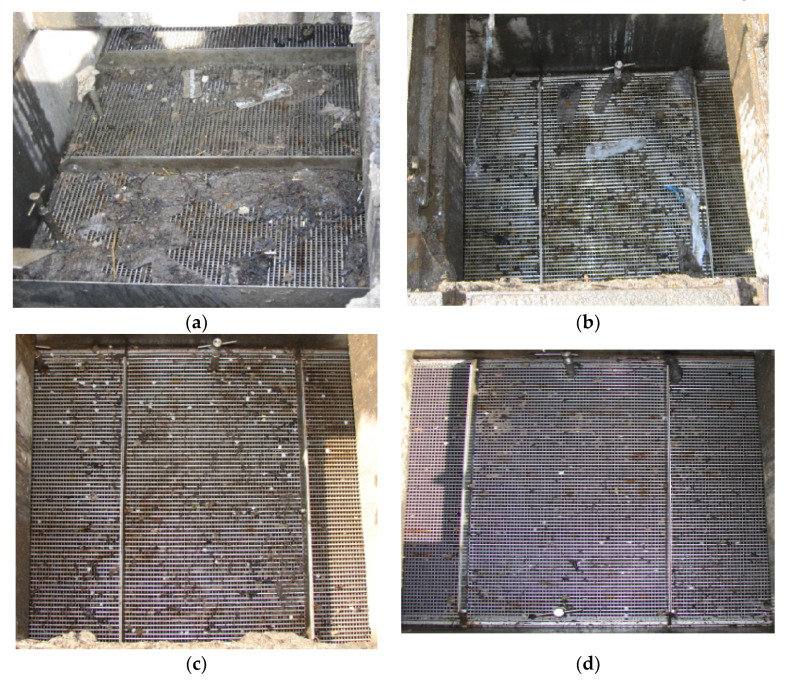
Phase 1: CSO screen conditions on site 1 in an urban area in West Yorkshire. (**a**) Unclean condition; (**b**) first level of cleaning; (**c**) second level of cleaning; (**d**) fully clean.

The first field trial site was located under a public highway located in West Yorkshire, as shown in Figure 5. This CSO chamber contained a 820 × 3380 mm^2^ screen. As can be seen from Figure 5, this CSO chamber can be accessed through three manhole lids and the sensor was placed on the top of the middle access as a major part of the screen was underneath it. The four screen conditions for this site are illustrated in Figure 6.

**Figure 7:**
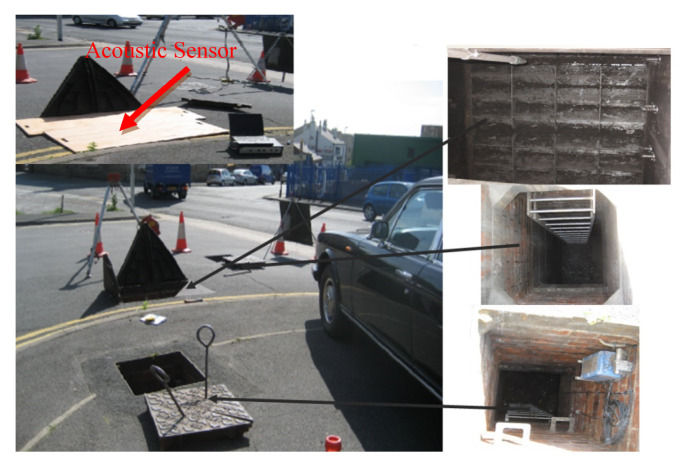
Phase 1: Field trial site 2 in West Yorkshire.

**Figure 8:**
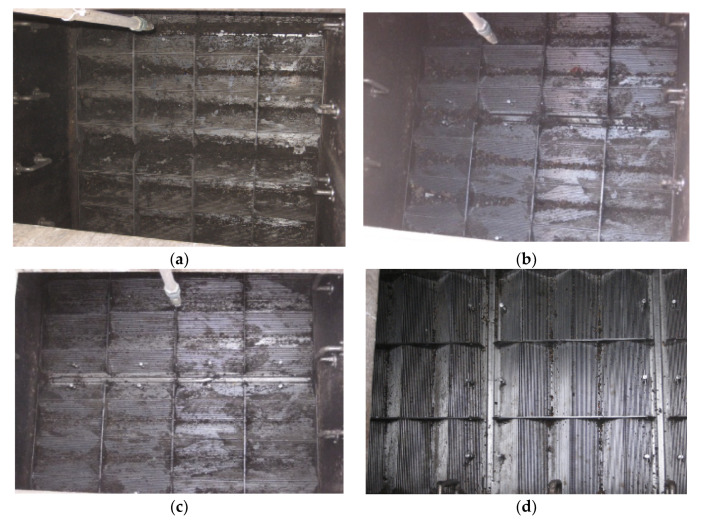
Phase 1: CSO screen conditions on site 2 in West Yorkshire. (**a**) Unclean condition; (**b**) first level of cleaning; (**c**) second level of cleaning; (**d**) fully clean.

The second field trial site was performed in a private highway. The section of land where the CSO was situated is privately owned and forms the main entrance to a garage and other businesses, as shown in Figure 7. The size of the CSO screen was 2300 × 4125 mm^2^, which was twice the size of the previous screen. Figure 7 depicts the site topology, interior and exterior of the CSO chamber, and the location where the sensor was installed. The four conditions of the CSO screen are shown in Figure 8 to give the indication of level of the cleanliness of the CSO screen: from unclean condition to fully clean condition.

**Figure 9:**
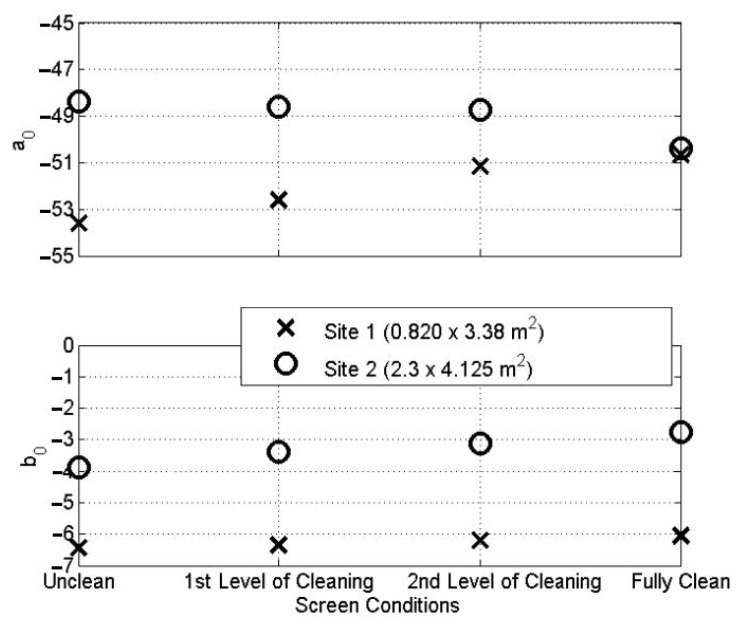
Results from phase 1 field trials on sites 1 and 2.

In both field trials, each of the test conditions was recorded five times for validation purposes. The average of the sound pressure level data was calculated and curved, fitted using the LMS method (Equations (3)–(5)). The coefficients *b*_0_ and *a*_0_ were estimated and plotted for the four CSO screen conditions, as shown in Figure 9. For site 1, the value of *b_0_* gradually increased from −6.5 for blinded condition to −6.0 for fully cleaned condition in the first field trial. This corresponds to an 8% increase in the value of *b*_0_ from the case of the chamber with a fully blinded screen to that with a fully clean screen. For site 2 with a larger screen size, the values of *b*_0_ are higher compared to those measured on site 1 with the smaller screen size. For site 2 experiments, the value of *b*_0_ increases from −3.9 for the case with a fully blinded screen to −2.8 for cases with a fully cleaned screen, which constituted a 39% change. The results from the two sites confirm that the smaller absolute value of the coefficient *b*_0_ corresponds to a cleaner CSO screen condition, whereas a larger absolute value indicates the CSO screen is likely to be blocked by debris, which absorbs more sound, resulting in less acoustic reflections. These results also confirm that a change in the screen condition is clearly detectable acoustically. The proposed acoustic method seems more sensitive in the case of a CSO screen, which is larger in size. This makes physical sense because the larger the screen area, the larger the potential change in the acoustic absorption in the chamber which a screen with deposits is likely to provide. This is reflected in a larger change in the reverberation time and absolute value of the coefficient *b*_0_.

### 3.2. Phase 2 Longer Term Experiments and Results

**Figure 10:**
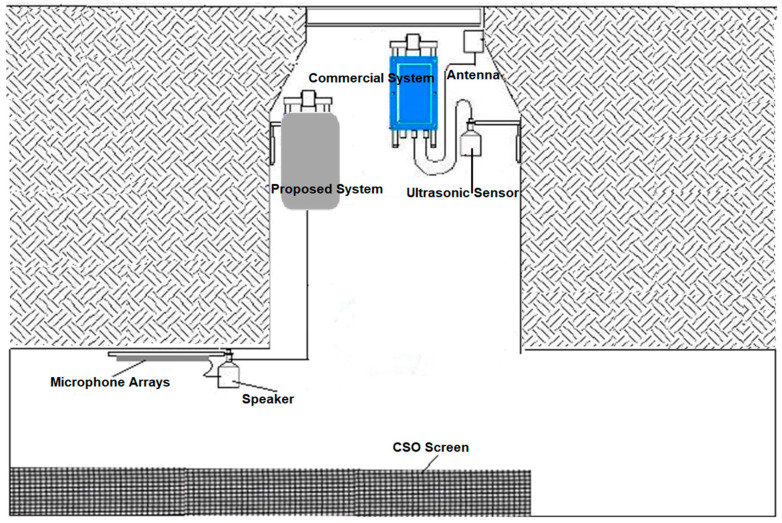
The installation of proposed acoustic sensor system for phase 2 experiments.

For the phase 2 field trial, a durable, intrinsically safe prototype of the acoustic instrument was developed to be left in the harsh conditions of the CSO chamber over a period of six months. This low-cost instrument was used to determine the dependence of the acoustic signature on a range of flow and screen conditions. This instrument can be linked to the existing telemetry, i.e., Hawkeye [29] technology for water level measurement. These two technologies can be combined to broadcast the recorded water level and acoustic data wirelessly. The acoustic and water level data can then be compared to determine the dependence of the coefficients *a*_0_ and *b*_0_ on the flow conditions in the CSO chamber. Photographs of the screen condition studied in phase 2 experiments were obtained from regular manual inspection, which was carried out every two weeks. Figure 10 shows schematically the setup for the installed screen sensor system and existing commercial Hawkeye ultrasonic water level metering system in the CSO chamber. These experiments enabled an evaluation of the reliability of the developed acoustic instrument to understand better its capabilities of operating in CSO chambers of various configurations under realistic operating conditions.

**Figure 11:**
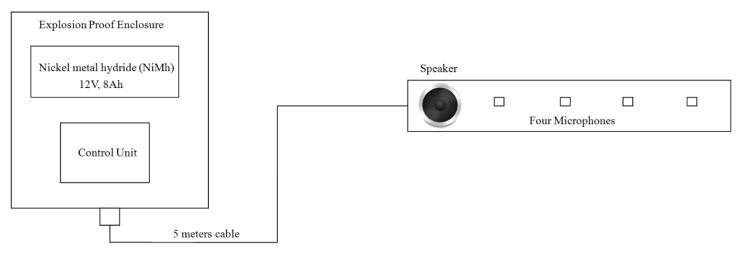
Schematic diagram of the acoustic sensor in the chamber.

**Figure 12:**
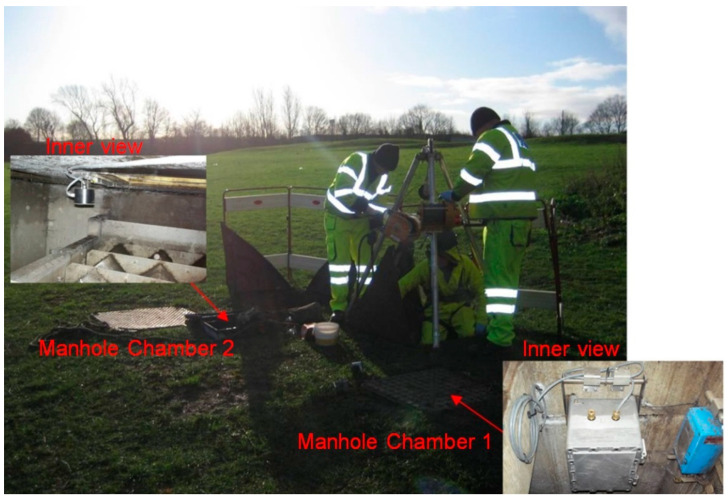
Practical implementation of the proposed acoustic system used in phase 2 field experiments.

The system used in phase 2 experiments consisted of an explosion-proof enclosure, which contained a control unit, power supply, microphone array and a speaker, as shown in Figure 11 and Figure 12. In order to minimise the risk of electrically generated sparks, the data acquisition unit, speaker, power amplifier and a 12 V, 8 Ah battery pack (nickel metal hydride, NiMh) were placed in an ATEX-certified explosion-proof box (EExd) manufactured by JCE Europe (EJB3A certification number CESIATEX004U). The speaker (Visaton 4 Ohm, model no: FRS 8, part no: 431-8686.) and the microphone array (Knowles Acoustics, model no: SPM0208HE5) remained outside the enclosure. These were connected to the electronic module in the explosion-proof enclosure via a 10-way weather-proof screened cable. The cable from the speaker and microphone module was made into the enclosure via an ATEX-certified EExd cable gland manufactured by Remora. In order to prevent the battery cell polarity reversal or reverse charging by another cell in the same battery, the guideline in British Standard (EN 60079-1:2007) was used. Shunt diodes (model: 1N4001S) were installed and connected to the battery cells to limit the reverse polarity voltage across each battery cell. Meanwhile, to prevent the inadvertent charging of a battery by other voltage sources in the enclosure, a pair of blocking diodes was used. 

The power supply lines were regulated electronically to 3.3 V for the control unit and microphone array and to 12 V for the power amplifier. These units were implemented on the same circuit board. In order to reduce the risk of a surge in the current in the external cable due to a malfunction in the electronics module, Zener barriers were placed on the control unit’s board, limiting the voltage going to the speaker to 12 volts (c, manufacture no: BZX79-C12) and the voltage going to the microphone array to 5.6 volts (Fairchild, manufacture no: 1N5232B, Fairchild Semiconductor International, Inc., San Jose, CA, USA). The maximum current drawn on the speaker was limited to 250 mA. In addition, both voltage regulators had short circuit protection to prevent excessive currents from reaching the microphone array and the speaker in case of malfunction of the system. If a short circuit occurred, then the circuit would be instantaneously disconnected from the power supply.

The speaker was hermetically sealed in an airtight plastic cylinder as shown in Figure 12. The part of the speaker diaphragm exposed to the atmosphere was free from any electrical wires and connections that could cause a shortcut when exposed to debris or water. The microphone array electronics were sealed in an airtight plastic enclosure. The acoustically sensitive parts of the microphones were vented to the atmosphere. All the connections between the parts of the system were made waterproof in order to prevent the water vapours present in the CSO chamber from condensing and short circuiting the electronics, causing a spark. 

Figure 12 also shows the location of the field trial site. As can be seen, it was located within a grassed recreation ground in West Yorkshire. The CSO chamber on this site was equipped with a static peak screen with dimensions of 505 × 1480 mm^2^ and a Hawkeye telemetry system [29,30] to measure the water level. This chamber could be accessed via two manholes, as shown in Figure 12. An ATEX-approved enclosure, which contained the data acquisition electronic devices, was installed in chamber 1, while the speaker and microphone arrays sensor bar were mounted in the centre position of the CSO screen within chamber 2. The sensor was programmed to carry out measurements and store data in the form of audio files at two-hour intervals. The CSO chamber was visited once every two weeks. During these visits, the conditions of the equipment were thoroughly checked and the SD memory card was replaced. The analysis of the data collected on the SD card was carried out back in the laboratory to study the consistency in the sensor operation and changes in the chamber over the measurement period.

**Figure 13:**
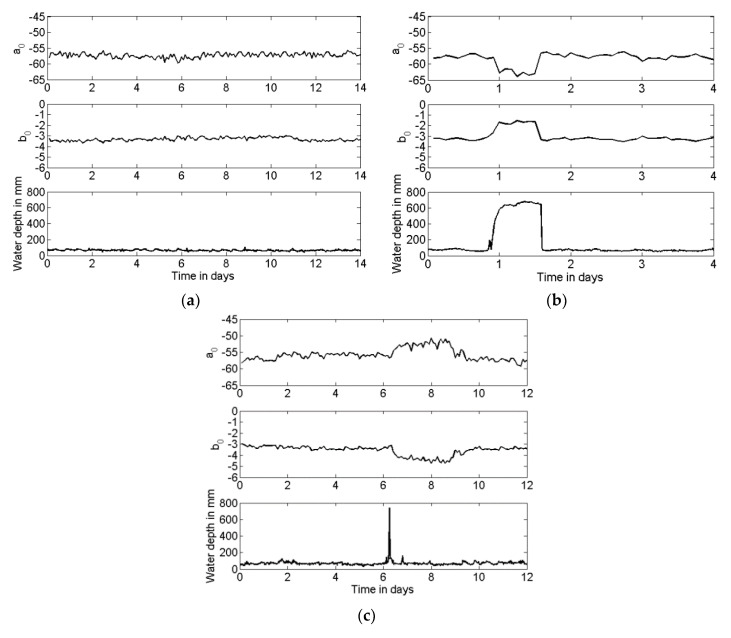
Examples of data collected in phase 2 over a six-month period: (**a**) ambient dry weather flow; (**b**) a longer term storm event which does not result in a change in screen condition; (**c**) a short-term storm event which resulted in a change in screen condition.

By examining of the conditions of the screen taken over a six-month period, it can be noticed that the CSO screen condition did not change significantly. This is reflected in a relatively small variation in the absolute values of the coefficients *a*_0_ and *b*_0_, except the times when the water level in the chamber changed considerably, affecting the screen condition. Some interesting observations from the change in the values of the coefficients *a*_0_ and *b*_0_ can be made. This change is illustrated in Figure 13, which shows examples of the time histories of the coefficients *a*_0_ and *b*_0_ measured over the six-month period. This figure also presents the water depth data, which were recorded at 15-min intervals with the Hawkeye telemetry system. The water level data are helpful to interpret the observed behaviour of the coefficients *a*_0_ and *b*_0_. During a 14-day measurement period which started on the 18th of January, there was not much rain around the instrumented site. Therefore, the ambient (dry weather flow) water level varied little between 70 mm and 100 mm (see Figure 13a). This is reflected in relatively constant values of the coefficients *a*_0_ and *b*_0_, which did not change by more than ±2.5% from their averages. This level of variation in the value of the coefficients *a*_0_ and *b*_0_ was a typical measure of the reproducibility of the proposed acoustic sensor.

On the 23rd of March, there was a storm event which caused a substantial rise in the water level in the chamber, up to approximately 700 mm for a period of 12 h (see Figure 13b). This level variation was clearly detected with the corresponding changes in the values of coefficients *a*_0_ and *b*_0_. When the water level in the chamber increased, the volume of the chamber was reduced. This resulted in a 5 dB decrease in the sound pressure level, which was reflected in the 5 dB decrease in the value of the coefficient *a*_0_ (see Figure 13b). As the level of water in the chamber increased, its volume reduced. Parts of the chamber floor and walls became covered by water so that the surface absorption in the chamber reduced and resulted in a longer reverberation time. The attenuation in the chamber was reduced, which is reflected in a 36% smaller absolute value of *b*_0_, as illustrated in Figure 13b. The values of these coefficients returned to the ambient as soon as the water level in the chamber dropped to the usual dry weather flow level. There was no evidence of any debris on the screen when the chamber was visited 12 days after the storm event for a routine check and battery replacement. Before and after this storm event, the variability in the value of the coefficients *a*_0_ and *b*_0_ was found to be relatively small and limited to the aforementioned value of ±2.5%. 

During the six-month measurement period, there were cases when the rise in the water level was a short-term event which deposited some debris on the screen. This type of event is illustrated in Figure 13c, which presents data recorded for 10 consequent days starting from the 1st of June. On day six of this recording, there was a rapid 700–800 mm rise in the water level due to a storm event. This event was relatively short and the rise in the water level was not captured with the acoustic sensor. However, some debris deposited on the screen by water was detected by the 30% increase in the absolute value of the coefficient *b*_0_ and 5 dB decrease in the value of coefficient *a*_0_. This change was substantial in comparison with the sensor reproducibility and clearly detectable acoustically. The results presented in Figure 13c also suggest that the screen condition naturally cleared after day nine. After that day, the two coefficients returned to their ambient values, as shown in Figure 13c. This was confirmed with the visual observation made on the 15th of June when the chamber was visited for regular checks. By that time, the debris deposited on the screen could have dried out and dropped off naturally, leaving the screen in a relatively clean condition.

## 4. Conclusions

In this paper, the feasibility of novel acoustic instrumentation to detect changes in the combined sewer overflow (CSO) screen condition was studied. The results obtained from two field trials (phases 1 and 2) deployed in urban residential areas provide an informed decision when the screen needs to be cleaned. The controlled field experiments in phase 1 suggest that the change between the clean and fully blinded CSO screen conditions is detectable with the proposed acoustic method. In the case of the smaller screen (site 1), this change was reflected in the 8% increase in the absolute value of the coefficient *b*_0_ in Equation (3). In the case of the larger screen, the corresponding increase in the absolute value of this coefficient was 39%. These changes are clearly detectable with the proposed acoustic method. This acoustic instrument, installed to monitor a plurality of types of CSO, will require initial calibration—a straightforward process which can be automated and completed remotely.

In phase 2, the data from the acoustic experiment in the field were compared against the Hawkeye water level data obtained in the same CSO chamber simultaneously, albeit at a different sampling interval. The acoustic sensor head worked properly over a period of six months. The reproducibility of the acoustic sensor data during the dry weather flow period was found within ±2.5%. This level of repeatability, together with obtained experimental evidence, suggest that the system can reliably detect changes in the conditions in the CSO screen which were caused by a rapid change in the water level. A deposition of debris on the CSO screen by a short-term storm event caused a 30% increase in the absolute value of coefficient *b*_0_ in Equation (3) and a 5 dB reduction in coefficient *a*_0_. These coefficients have recovered to their ambient values after the screen returned naturally to its original condition. 

The results also show that the sensor can detect water level changes which occur over a period of time, which is longer than the sensor sampling interval. A significant increase in the water level causes a measurable drop in the sound pressure level in the chamber and reduction in the acoustic attenuation. These results make physical sense. It was found that the acoustic sensor performance returns to normal after the level of water drops to its ambient dry weather conditions. 

The speed of the acoustic measurements (a few seconds) and sensor’s ability to quantify the screen condition remotely have the potential to be at least two orders of magnitude less costly than traditional site visit and visual inspection methods. The results from phase 2 experiments show that the screen condition can naturally recover after a short-term storm event, which usually causes some debris deposition. This information is novel as it has not been observed before. This implies that the deployment of such sensors can help to reduce unnecessary site inspection visits, resulting in a reduction of operational costs and associated environmental impact. The proposed instrument can be improved further by pairing it with an energy harvesting system (e.g., solar or wind) and integrating it with the wireless network, e.g., the Hawkeye system.

## Data Availability

The data presented in this study are available on request from the corresponding author. The data are not publicly available due to the Confidentiality and Non-disclosure Agreement with the funders.
